# Photonic spin Hall effect enabled refractive index sensor using weak measurements

**DOI:** 10.1038/s41598-018-19713-3

**Published:** 2018-01-19

**Authors:** Xinxing Zhou, Lijuan Sheng, Xiaohui Ling

**Affiliations:** 1 Synergetic Innovation Center for Quantum Effects and Applications, College of Physics and Information Science, Hunan Normal University, Changsha, 410081 China; 2 Hunan Provincial Key Laboratory of Intelligent Information Processing and Application, College of Physics and Electronic Engineering, Hengyang Normal University, Hengyang, 421002 China

## Abstract

In this work, we theoretically propose an optical biosensor (consists of a BK7 glass, a metal film, and a graphene sheet) based on photonic spin Hall effect (SHE). We establish a quantitative relationship between the spin-dependent shift in photonic SHE and the refractive index of sensing medium. It is found that, by considering the surface plasmon resonance effect, the refractive index variations owing to the adsorption of biomolecules in sensing medium can effectively change the spin-dependent displacements. Remarkably, using the weak measurement method, this tiny spin-dependent shifts can be detected with a desirable accuracy so that the corresponding biomolecules concentration can be determined.

## Introduction

The spin Hall effect (SHE) manifests itself as the splitting of spin-up and spin-down electrons, inducing transverse spin currents perpendicular to the applied electric field direction^1–3^. Recently, an interesting phenomenon called photonic SHE has been attracting more and more attention^4–6^. The photonic SHE can be seen as an optical analogy of SHE in electronic system where the spin photons play the role of the spin electrons and the applied electric field is replaced by the refractive index gradient. The physical mechanism of the photonic SHE is attributed to an effective spin-orbit coupling between the spin (polarization) and the trajectory of light beam^7–9^. Generally, the spin-dependent splitting in photonic SHE is very tiny, leading to shifts too small to be detected directly. In 2008, thanks to the weak measurement method, the Kwiat’s group first observed the transverse shift of a light beam refracted from a glass prism^6^.

Until now, the photonic SHE has been widely studied in different physical systems such as optical physics^10–12^, metasurfaces^13,14^, high-energy physics^15,16^, plasmonics^17,18^, semiconductor physics^19^, and also in free-space^20^. Especially, with the presence of an external magnetic field, the quantized photonic SHE in graphene has been found^21^. However, most of these researches focus on the theoretical calculation and experimental measurement of photonic SHE. There are few studies on its practical application. In fact, the photonic SHE holds great potential for precision metrology, because the spin-dependent displacements in photonic SHE are sensitive to the physical parameter variations of the systems. Using the weak measurements, the tiny shift can be observed with the desirable accuracy leading to the corresponding physical parameters be determined. For example, the photonic SHE has been used for measuring the thickness of nanometal films^22^, identifying the graphene layers^23^, detecting the strength of axion coupling of topological insulators^24^, and estimating the optical rotation of chiral molecules^25^.

In this work, we theoretically propose an optical biosensor based on photonic SHE. This biosensor consists of a BK7 glass, an Au film, and a graphene sheet. In the sensing medium, a variation in biomolecules concentration will induce a local change in the refractive index near the graphene surface. We find that, by considering the surface plasmon resonance (SPR) effect, the spin-dependent shifts in photonic SHE are sensitive to the refractive index variations of sensing medium. The paper is orgonized as follows. At first, we consider the horizontal (H) polarization beam reflected at the interface between the sensor and the sensing medium. Here, the quantitative relationship between the spin-dependent splitting and the refractive index of sensing medium is obtained. Then, we theoretically analyze the spin-dependent splitting in biosensor by modulating the refractive index of sensing medium. Importantly, a signal enhancement technique known as weak measurement is theoretically proposed to observe this tiny shift. Finally, a conclusion is given.

## Theoretical Model

**Figure 1:**
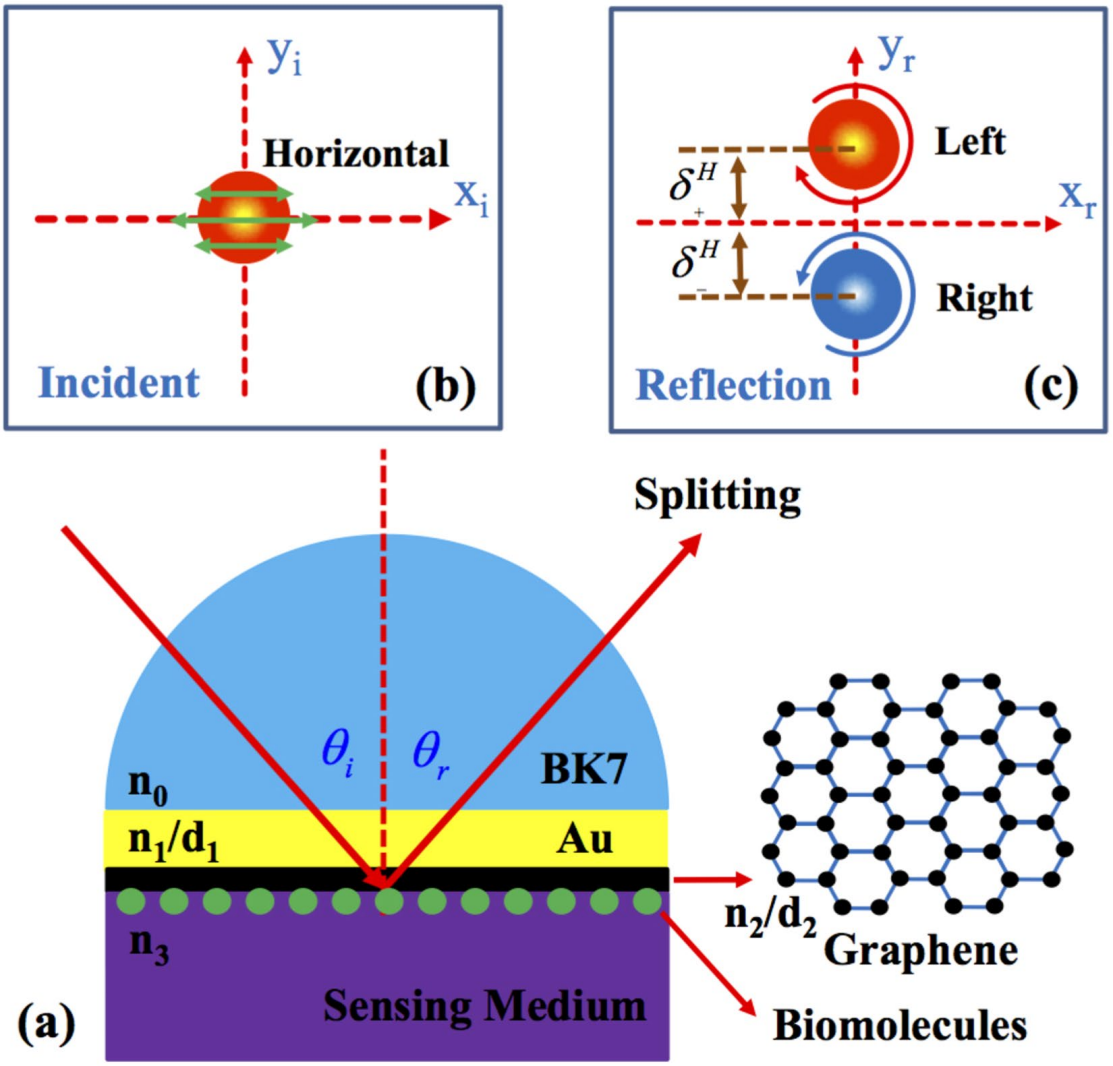
Schematic of the biosensor and the corresponding photonic SHE. (**a**) The physical structure of the biosensor. It is composed of a BK7 glass, an Au film, and a graphene sheet. The inset shows the atomic structure of graphene. (**b**) The intensity and polarization distribution of the incident light beam. (**c**) The intensity and polarization distribution of the reflected light beam. ${\delta }_{\pm }^{H}$ indicate the transverse (in y direction) shifts of left- or right-circularly polarized component. To make the splitting characteristics more noticeable, we have amplified the initial spin-dependent shifts ${\delta }_{\pm }^{H}$. *θ*_*i*_ and *θ*_*r*_ are the incident and reflected angle.

In this section, we first establish a general propagation model to describe the process of the light beam reflected at the sensor interface and study the corresponding spin-dependent splitting varying with the refractive index change of sensing medium. We only consider the case of H polarization beam, and the case of vertical (V) polarization beam can be analyzed in a similar way. The structure of the biosensor is composed of a BK7 glass, an Au film, and a graphene sheet [as shown in Fig. 1(a)]. The graphene film used here is based on the reason that it can adsorb biomolecules with unique carbon-based ring structures enabling a sensitive refractive index variation near the interface between the graphene and sensing medium^26,27^. Additionally, the surface to volume ratio of graphene is very high providing large surface area for exposing it to the surrounding so that it can adsorb the biomolecules with very high efficiency^28^. In this structure, the symbols *n*_0_, *n*_1_, *n*_2_, and *n*_3_ denote the corresponding refractive index of different materials. *d*_1_ and *d*_2_ are the thickness of Au film and graphene. Figure 1(b) shows the intensity distribution of incident light beam. After reflection, the photonic SHE appears and the incident linearly polarized beam will split into its left- and right-handed circularly polarized components [Fig. 1(c)]. It is noted that we only consider the transverse spin-dependent splitting.

At first, let us analyze the incident and reflected electric fields of light in this model. In the spin basis set, the angular spectrum can be expressed as ${\tilde{{\bf{E}}}}_{i}^{H}=({\tilde{{\bf{E}}}}_{i+}+{\tilde{{\bf{E}}}}_{i-})/\sqrt{2}$ and ${\tilde{{\bf{E}}}}_{i}^{V}=i({\tilde{{\bf{E}}}}_{i-}-{\tilde{{\bf{E}}}}_{i+})/\sqrt{2}$. The positive and negative signs in the supscripts represent left- and right-handed circularly polarized components. We consider a monochromatic Gaussian beam reflects at the sensor interface and its spectrum can be written as

1 $${\tilde{{\bf{E}}}}_{i\pm }=({{\bf{e}}}_{ix}\pm i{{\bf{e}}}_{iy})\frac{{w}_{0}}{\sqrt{2\pi }}\exp [-\frac{{w}_{0}^{2}({k}_{ix}^{2}+{k}_{iy}^{2})}{4}],$$

where *w*_0_ is the beam waist. To study the reflected field of light beam, we need to deduce the relationship between the incident and reflected fields. Through the coordinate rotation, we can calculate the reflected angular spectrum according to the relation ${\tilde{{\bf{E}}}}_{r}({k}_{rx},{k}_{ry})={{\bf{M}}}_{R}{\tilde{{\bf{E}}}}_{i}({k}_{ix},{k}_{iy})$. The above ***M***_*R*_ stands for^22^

2 $$[\begin{array}{l}{\tilde{{\bf{E}}}}_{r}^{H}\\ {\tilde{{\bf{E}}}}_{r}^{V}\end{array}]=[\begin{array}{cc}{r}_{p} & \frac{{k}_{ry}\,\cot \,{\theta }_{i}({r}_{p}+{r}_{s})}{{k}_{0}}\\ -\frac{{k}_{ry}\,\cot \,{\theta }_{i}({r}_{p}+{r}_{s})}{{k}_{0}} & {r}_{s}\end{array}]\,[\begin{array}{l}{\tilde{{\bf{E}}}}_{i}^{H}\\ {\tilde{{\bf{E}}}}_{i}^{V}\end{array}].$$

here, *r*_*p*_ and *r*_*s*_ are Fresnel reflection coefficients for H and V polarization states. *k*_0_ is the wave number in free space.

According to Eqs (1) and (2), we can first get the expression of reflected angular spectrum

3 $${\tilde{{\bf{E}}}}_{r}^{H}=\frac{{r}_{p}}{\sqrt{2}}[\exp (+i{k}_{ry}{{\rm{\Delta }}}_{r}^{H}){\tilde{{\bf{E}}}}_{r+}+\exp (-i{k}_{ry}{{\rm{\Delta }}}_{r}^{H}){\tilde{{\bf{E}}}}_{r-}].$$

here, ${{\rm{\Delta }}}_{r}^{H}=\mathrm{(1}+{r}_{s}/{r}_{p})\cot \,{\theta }_{i}/{k}_{0}$. It is known that the spin-orbit coupling is the intrinsic physical mechanism of the photonic SHE. We note that, in Eq. (3), the terms $\exp (\pm i{k}_{ry}{{\rm{\Delta }}}_{r}^{H})$ indicate the spin-orbit coupling terms in the case of H polarization. The spin-orbit coupling term comes from the transverse nature of the photon polarization where the polarizations associated with the plane-wave components of the light beam experience different rotations in order to satisfy the transversality after reflection^6,7^.

The calculation of the reflected beam shifts in photonic SHE requires the explicit solution of the boundary conditions at the biosensor interfaces. Thus, we need to know the generalized Fresnel reflection coefficients of this model. This relationship can be deduced from the 2 × 2 transmission matrix^29^:

4 $$N={T}_{01}{P}_{1}{T}_{12}{P}_{2}{T}_{23}{P}_{3}\mathrm{..}.{P}_{l-2}{T}_{l-\mathrm{2,}l-1}{P}_{l-1}{T}_{l-\mathrm{1,}l},$$

where

5 $${T}_{l-\mathrm{1,}l}=\frac{1}{{t}_{l-\mathrm{1,}l}}[\begin{array}{cc}1 & {r}_{l-\mathrm{1,}l}\\ {r}_{l-\mathrm{1,}l} & 1\end{array}],$$

describes the transformation matrix from (*l* − 1)-th to *l*-th layer, and

6 $${P}_{l}=[\begin{array}{cc}\exp (i{k}_{lz}{d}_{l}) & 0\\ 0 & \exp (-i{k}_{lz}{d}_{l})\end{array}],$$

represents the transmission matrix for *l*-th layer. Here, *d*_*l*_ is the thickness of *l*-th layer material, *r*_*l*−1,*l*_ and *t*_*l*−1,*l*_ are reflection and transmission coefficients at the interface between (*l* − 1)-th and *l*-th layer, respectively. For an arbitrary wave-vector component, we can obtain the Fresnel reflection coefficient of the layered nanostructures

7 $${r}_{p,s}=\frac{{N}_{21}}{{N}_{11}},$$

where *p* and *s* denote parallel and perpendicular polarizations, respectively. And the notations *N*_21_ and *N*_11_ are the matrix elements of Eq. (4). Here, the refractive index of sensing medium is coupled in the Fresnel reflection coefficient. To get the desired results, we should expand the Fresnel reflection coefficients around the central wave vector by making use of a Taylor series expansion based on the arbitrary angular spectrum component:

8 $${r}_{p,s}={r}_{p,s}({\theta }_{i})+\frac{\partial {r}_{p,s}}{\partial {\theta }_{i}}\frac{{k}_{ix}}{{k}_{0}}.$$

A sufficiently good approximation can be obtained when the Taylor series are confined to the zeroth order. Interestingly, by considering the in-plane wave vector component *k*_*ix*_, there exists the in-plane spin-dependent splitting^30^.

In this model, the photonic SHE is described for the left- and right-circularly polarized components undergoing transverse spin-dependent splitting. So the reflected field centroid should be determined. At any given plane *z*_*a*_ = *const*., the displacements of field centroid compared to the geometrical-optics prediction is given by^23^

9 $${\delta }_{\pm }^{H}=\frac{\int \int {\tilde{{\bf{E}}}}^{\ast }i{\partial }_{{{\bf{k}}}_{ry}}\tilde{{\bf{E}}}d{k}_{rx}d{k}_{ry}}{\int \int \tilde{{\bf{E}}}\ast \tilde{{\bf{E}}}d{k}_{rx}d{k}_{ry}}.$$

Finally, substituting Eq. (3) into Eq. (9), we can get the transverse spin-dependent displacements of photonic SHE in biosensor.

## Results and Discussion

In the above analysis, we have established the relationship between the refractive index of sensing medium and the spin-dependent splitting in photonic SHE. Next, we will discuss how the biosensor works. At first, we need to choose the suitable parameters of this structure (Fig. 1). Here, the refractive index of BK7 glass and Au film are chosen as *n*_0_ = 1.515 and ${n}_{1}=\sqrt{-10.4+1.4i}$ at 633 nm^31^. The thickness of Au is picked as 50 nm. The refractive index of graphene film is selected as *n*_2_ = 3.0 + 1.149*i* at 633 nm^32^. The thickness of the graphene film is about *d*_2_ = *m*Δ*d* in which *m* denotes the layer numbers and Δ*d* represents the thickness of single layer graphene (Δ*d* is about 0.34 nm)^32^. In fact, our biosensor is a four-layered nanostructure. So the parameter *l* from Eqs (4)–(6) is fixed to 3. In the sensing medium, a variation in biomolecules concentration will induce a local change in the refractive index *n*_3_. In this work, we consider a small refractive index variation of sensing medium Δ*n*_3_ = 0.005 (from 1.33 to 1.335). In the following, we will investigate how to detect this tiny refractive index change using the photonic SHE.

**Figure 2:**
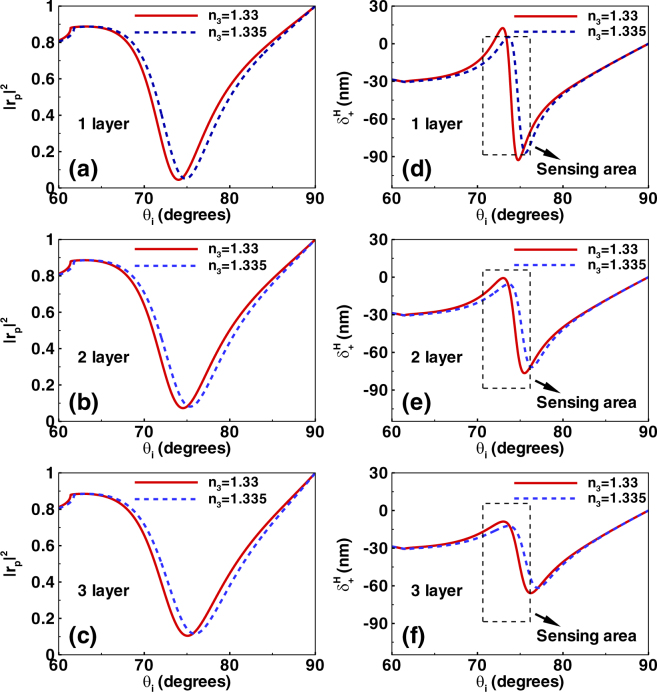
The reflectivity and the initial spin-dependent splitting in photonic SHE. (**a**–**c**) Show the reflectivity of light beam when the refractive index of sensing medium are chosen as *n*_3_ = 1.33 and 1.335. Here, the number of the graphene layers are 1, 2, and 3. The calculated parameters of the biosensor are discussed in the main text. The initial spin-dependent shifts of photonic SHE are described in (**d**–**f**). The range of incident angles are focused on 60° to 90°. Dotted boxes represent the sensing area in which the spin-dependent splitting are sensitive to the refractive index change of sensing medium.

Figure 2 shows the reflectivity and the spin-dependent displacement changing with the refractive index *n*_3_, the number of the graphene layers, and the incident angle *θ*_*i*_. Here, the reflectivity is defined as |*r*_*p*_|^2^ [as shown in Fig. 1(a)–(c)]. We can see that the reflectivity undergoes a sharp decreasing when the angle of incidence is near about 75° (resonance angle). This phenomenon is attributed to SPR. It is noted that the resonance angle experience a small shift when the refractive index of sensing medium vary from *n*_3_ = 1.33 to *n*_3_ = 1.335. We can detect the refractive index change by observing this resonance angle offset. This is the usual idea of the SPR-based biosensor^26^. However, the biosensor discussed here is from a different perspective (combining with the photonic SHE).

Figure 2(d)–(f) show the photonic SHE changing with the refractive index of sensing medium, the number of the graphene layers, and the incident angle. When the incident angle is near the resonance angle, there exist a dramatic variation of the spin-dependent splitting by choosing the refractive index of sensing medium with *n*_3_ = 1.33 and *n*_3_ = 1.335. As shown in the picture, a sensing area can be established to detect the sensing medium changes by observing the photonic SHE. Here, for a fixed incident angle, we can obtain two values of spin-dependent shifts corresponding to two values of refractive index *n*_3_. Therefore, we can deduce the properties of sensing medium by indirectly measuring the spin-dependent displacements of photonic SHE. Note that the spin-dependent splitting is not sensitive to the small change of the refractive index of the sensing medium when the incident angle leaves the sensing area. Generally, the larger the refractive index difference, the easier the measurement. However, we focus our attention on the detection of tiny refractive index variations.

**Figure 3:**
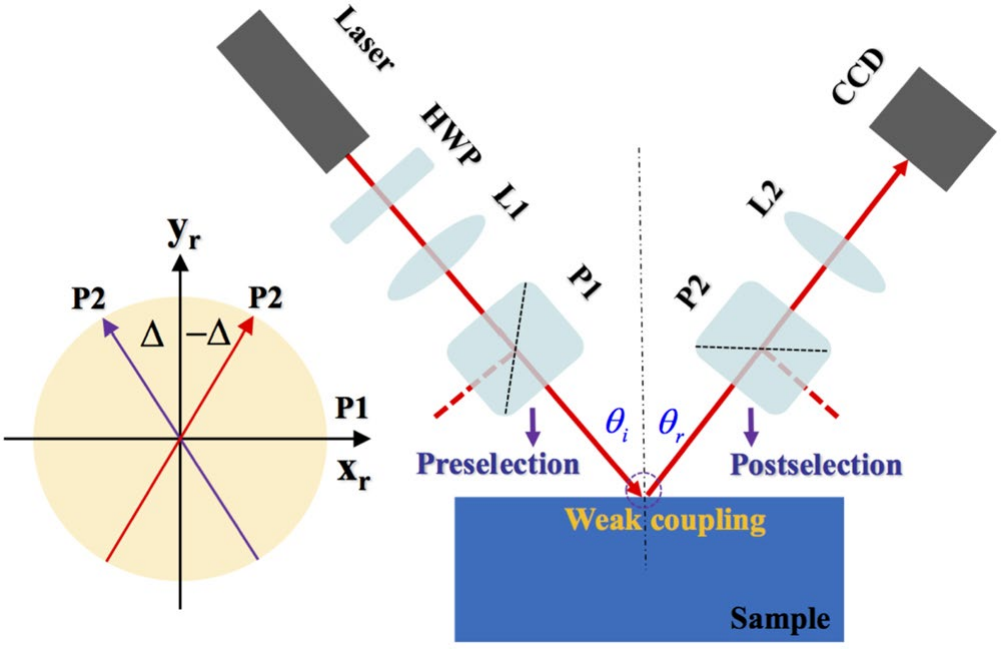
The proposed experimental setup: Sample, the biosensor composed of a BK7 glass, an Au film, and a graphene sheet. L1 and L2: lenses with effective focal length. HWP, half-wave plate (for adjusting the intensity). P1 and P2, Glan Laser polarizers. CCD, charge-coupled device for detecting the signal. The light source is a linearly polarized He-Ne laser. Here, the weak measurements is divided into three steps: preselection, weak coupling, and postselection. The inset represents that the angle between the transmission axes of P1 and P2 is 90° ± Δ.

In fact, the photonic SHE in this biosensor is too small to be observed directly. Here we theoretically propose a signal enhancement technique called weak measurement^33^ to measure this spin-dependent splitting. The weak measurement technology based on preselection and postselection states is a promising method for in investigating fundamental questions of quantum mechanics^34,35^ and holds great potential for precision measurement^36–38^. The possible experimental setup is shown in Fig. 3. The incident beam is focused by the lens (L1) and is preselected in the H polarization with P1, and then it undergoes postselected in the polarization state with **V** = sinΔ**e**_*rx*_ + cosΔ**e**_*ry*_ by P2. The two polarization states are nearly perpendicular to each other with an angle of 90° ± Δ. There exists two types of amplified factors *A*_*w*_ (weak value amplified factor) and *F* (propagation amplified factor), which gives an enhanced displacement proportional to initial one ${\delta }_{w}^{H}={A}_{w}^{{mod}}{\delta }_{\pm }^{H}$, where ${A}_{w}^{{mod}}=|{A}_{w}|F$. As is well known, the small overlap between the initial and final states will results in a loss of signal. In fact, the technical noise can be significantly reduced by the weak value amplification^6^.

**Figure 4:**
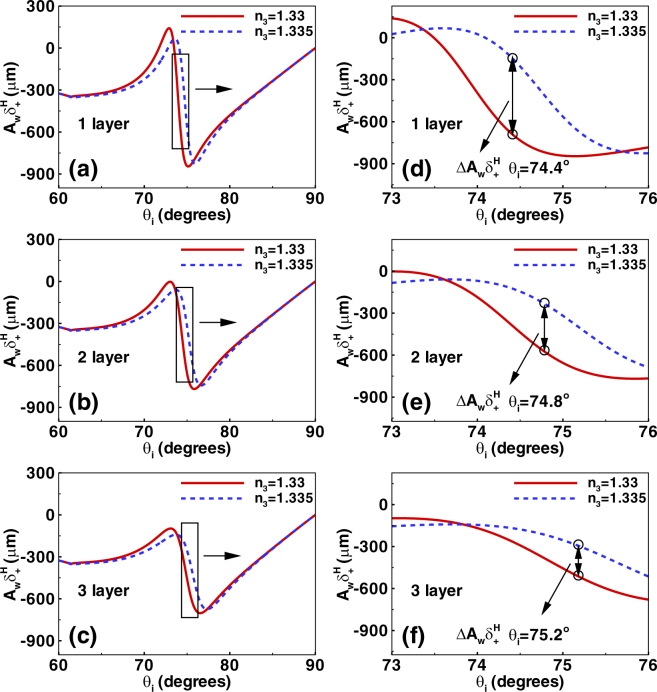
The amplified spin-dependent shifts of photonic SHE by using the weak measurement method. (**a**–**c**) Show amplified displacements in biosensor by considering the refractive index of sensing medium with *n*_3_ = 1.33 and 1.335. The graphene are also chosen as one layer, two layers, and three layers. (**d**–**f**) Describe the transverse shifts of different incident angles ranging from 73° to 76°. There exists a maximum deviation point where the relative amplified shifts deviation ${\rm{\Delta }}{A}_{w}{\delta }_{+}^{H}$ represent the maximum value. The calculated parameters of biosensor are discussed in the main text.

Combining with the Eqs (3) and (9), the relevant amplitude of the reflected field at a given plane *z*_*r*_ can be obtained as ${\bf{V}}\cdot {{\bf{E}}}_{r}^{H}$ allowing for calculation of amplified displacement which is the output of this sensing system. Here, we choose Δ = 0.5° and *z*_*r*_ = 220 mm. The amplified shifts of photonic SHE are shown in Fig. 4. Here, we choose the number of the graphene layers as 1, 2, and 3. The range of the incident angle is from 60° to 90°. Figure 4(a)–(c) describe the amplified shifts corresponding to the initial spin-dependent splitting from Fig. 2(d)–(f). We find that the amplified shifts reach about hundreds of microns which can be measured directly. So we can determine the refractive index change of sensing medium by detecting the amplified displacements of photonic SHE in biosensor.

From Fig. 4(a)–(c), we find that it is hard to distinguish the transverse shifts of the different refractive index of sensing medium (*n*_3_ = 1.33 or 1.335) from 60° to 90°. So we focus our attention on the transverse displacements in a small range of incident angle (from 73° to 76°) to obtain desired results [Fig. 4(d)–(f)]. Remarkably, there exist a significant deviation of the amplified shifts in photonic SHE when refractive index of sensing medium are *n*_3_ = 1.33 and 1.335. In order to improve the accuracy of the sensor, we should select the maximum deviation point where the relative amplified shifts deviation ${\rm{\Delta }}{A}_{w}{\delta }_{+}^{H}={A}_{w}{\delta }_{+}^{H,1.335}-{A}_{w}{\delta }_{+}^{H,1.33}$ represent the maximum value. For example, in Fig. 4(d), the relative amplified shifts deviation ${\rm{\Delta }}{A}_{w}{\delta }_{+}^{H}$ reaches about 544 *μm* when the maximum deviation point is about *θ*_*i*_ = 74.4°. This is a large shift which will make the biosensor more accurate. We also define the sensitivity of biosensor as ${S}_{n}={\rm{\Delta }}{A}_{w}{\delta }_{+}^{H}/{\rm{\Delta }}{n}_{3}$. Under this condition, the sensitivity of biosensor is about 1.088 × 10^5^ *μm*/RIU. It is found that the high sensitivity can be derived from large optical shift offset at the same refractive index variation. Similarly, the maximum deviation points are *θ*_*i*_ = 74.8° and *θ*_*i*_ = 75.2° when the number of the graphene layers are chosen as 2 and 3 [Fig. 4(e) and (f)]. Therefore, we can detect the biomolecules concentration by observing the amplified shifts of photonic SHE in this biosensor. From the above analysis, we can conclude that the optimal number of graphene layer of this optical sensor is one (having the relatively large deviation of amplified shifts corresponding to the same variation of refractive index).

**Figure 5:**
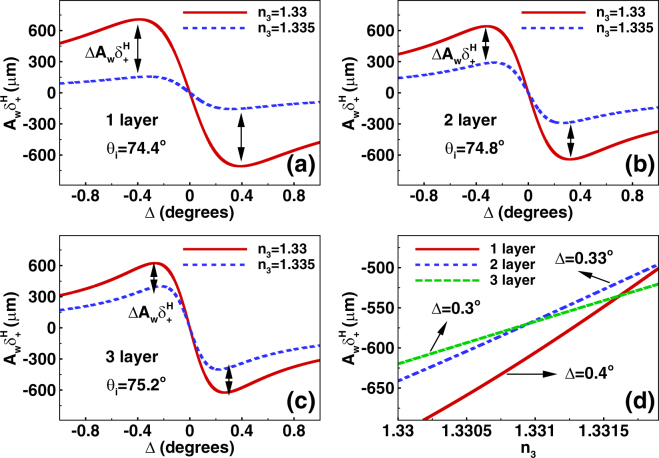
Determining the optimal amplified angle and the number of graphene layer in this optical sensor. (**a**–**c**) Denote the amplified spin-dependent displacements changing with the amplified angle Δ. The refractive index of sensing medium are chosen as *n*_3_ = 1.33 and 1.335, and the graphene are selected as one layer, two layers, and three layers. The incident angles are fix to the optimal values discussed in the above picture. Here, the optimal amplified angles for obtaining the maximum shift deviation are Δ = 0.4°, 0.33°, and 0.3°. (**d**) Show the amplified spin-dependent shifts varying with the refractive index of sensing medium. The solid, dashed, and dotted curves describe the beam shifts corresponding to the different optimal amplified angles.

We also investigate the amplified spin-dependent splitting changing with the amplified angle Δ, as shown in Fig. 4(a)–(c). Here, the refractive index of sensing medium are chosen as *n*_3_ = 1.33 and 1.335, and the graphene are selected as one layer, two layers, and three layers. We fix the incident angles to the above optimal points (with maximum deviation of the amplified shifts in Fig. 4). It is found that the maximum deviation of the amplified shifts can also be obtained by choosing the optimal amplified angles (Δ = 0.4°, 0.33°, and 0.3° corresponding to the different numbers of graphene layer). Thus, we can get high sensitivity of this optical biosensor by modulating the amplified angles. Figure 5(d) describes the amplified displacements varying with the refractive index of sensing medium. The amplified angles are chosen as the optimal values. We can find that the amplified shifts are proportional to the biomolecules concentration for the tiny refractive index variation. It is noted that the slope of the curve represents the sensitivity of the biosensor. We find that the slope of the solid line (corresponding to the one layer graphene) is larger than the others, also showing that the optimal number of graphene layer of this optical sensor is one.

How to improve the precision of the optical sensor, especially for the measurement of objects with tiny refractive index variation, is a very important issue for the developing of high performance sensor. The key point is to design a sensor with high signal-to-noise ratio (SNR). In fact, the weak measurement can be an effective method for improving the precision of measurement because the detected signal is amplified while the technical noise is suppressed^6^, therefore the SNR can be significantly enhanced. Recently, an improved SPR sensor based on appropriate preselection and postselection states was proposed^39^, showing nearly one order of precision enhancement compared with the conventional prism-coupler based SPR sensor. In the present work, our proposed optical sensor is also based on the weak measurement technique, so that the technical noise can be sufficiently suppressed leading to the imporvement of SNR. Additionally, the propagation amplified factor *F* mentioned above can also increase the SNR in the weak measurement system^40^. Therefore, the precision of optical sensor designed here can be further improved compared with the normal SPR sensor.

## Conclusion

In conclusion, we have investigated the photonic SHE in a proposed four-layered nanostructure. By considering the SPR effect, we have found that the spin-dependent splitting in photonic SHE are sensitive to the refractive index change of sensing medium, which makes this nanostructure a good candidate for biosensor. Importantly, by combining with the weak measurement method, the initial tiny spin-dependent shifts in biosensor can be detected with the desirable accuracy so that the corresponding biomolecules concentration can be determined. These findings provide a practical application for photonic SHE and open the possibility of developing spin-based nanophotonic device.

### Data Availability

No datasets were generated or analysed during the current study.
